# A systematic assessment of the demand for HTA hub services in Asia

**DOI:** 10.1017/S0266462325000236

**Published:** 2025-04-25

**Authors:** Julie Hoang, Jeffrey Sine, Sweta Saxena, Christian Suharlim

**Affiliations:** 1 Management Sciences for Health, Medford, MA, USA; 2 United States Agency for International Development, Washington, DC, USA

**Keywords:** capacity strengthening, technology assessment, health, Asia

## Abstract

**Objectives:**

This assessment aimed to identify the degree and parameters of demand for support from HTAsiaLink, the Asia regional health technology assessment (HTA) hub, for HTA ecosystem development.

**Methods:**

A sequential, exploratory, mixed-method design was implemented, starting with a literature review to define the Asia region’s HTA landscape. Then an online survey was sent to 125 Asia-focused HTA practitioners and support organizations to obtain their thoughts on HTA development needs and how a regional hub could serve them. Finally, fifty purposively selected key informants representing government HTA agencies in Asia, funding partner organizations, philanthropic foundations, global HTA support, and regional HTA hub organizations were invited to participate in semi-structured interviews. Nineteen Asian countries and territories were represented in documents reviewed. Twenty-five recipients from ten Asian countries and territories responded to the survey, and twenty-eight individuals from eight Asian countries and territories plus eight international organizations participated in interviews.

**Results:**

Identified needs include support to fill HTA human resources gaps, strengthen the capacity of the existing HTA workforce, produce HTA public goods, improve harmonization within and across country systems, and strengthen political will. Other important considerations include the need to adapt the hub’s purpose to an expanding role and adopt sustainable financing approaches accordingly.

**Conclusion:**

Demand for an HTA hub in Asia is high, including to support HTA technical, deliberative processes, and institutional capacity strengthening. Findings underscore the importance of both conducting HTAs and fostering demand for HTA output. HTAsiaLink is recognized as well-positioned to play an expanded support role to address these needs.

## Introduction

Health technology assessment (HTA) is a pivotal tool for healthcare and health systems priority setting globally. In Asia, as in other regions, the volume of HTAs is increasing, though the processes for using HTA output in decision-making and HTA institutionalization may lag. Suharlim et al. (1) identified key drivers to the introduction and institutionalization of HTA, highlighting the significance of increased collaboration among countries. Collaboration includes sharing good practices and engagement in international networks (1).

Understanding these drivers is key to designing approaches to support countries as they institutionalize HTA as a mainstream tool for health sector decision-making about resource use and priority setting. Global and regional collaboration provides countries with opportunities to support HTA ecosystem development. HTA hubs facilitate collaboration by encouraging member participation. Membership in international HTA organizations is one of eighteen progress milestones Kumar et al. (2) used to map national HTA systems development journeys. In Asia, the degree of participation in HTA forums varies across countries and territories (2).

Hubs exist in various forms; they are often vehicles through which networking and knowledge sharing occur (3). Hubs can be complex organizations when they include dimensions of dialogue forums, capacity strengthening, and technical assistance provision. Adapting from Evers’ definition of hubs (3) and for the purposes of this study, we define an HTA hub as an organization whose primary purpose is to promote and support improvements in the environment for conducting HTAs and using HTA results for health sector decision-making. Notable regional HTA hubs are the Health Technology Assessment Network of the Americas (RedETSA), with twenty-one countries and forty-two institutional members, and the network established by the European Union (EU), formerly known as the European Network for Health Technology Assessments (EUnetHTA), with thirty countries and eighty-three institutional members. HTA hubs seek to add value to processes that advance HTA practice and its institutionalization by increasing collaboration among members and supporting networking, knowledge exchanges, and capacity strengthening.

In 2011, the Asia region marked a significant milestone in HTA development with the establishment of HTAsiaLink, a network that provides a close and accessible resource for member organizations. What initially began as a platform for supporting early career researcher development and for sharing research findings, HTAsiaLink’s role has evolved. It is a conduit for propagating awareness about the utility of HTA in health sector priority setting (4), mostly through convening annual conferences on regional HTA activities and development. Despite regional advancements in HTA knowledge sharing, there is little information regarding perspectives about the roles an HTA hub in Asia should fulfill.

To address this information gap, the United States Agency for International Development (USAID) commissioned the USAID Medicines, Technologies, and Pharmaceutical Services (MTaPS) Program – a consortium led by Management Sciences for Health (MSH) – in 2022, to investigate the dimensions of demand for HTA hub support in Asia. This paper presents results from a systematic assessment of the demand, with the intention of providing insights for supporting further development of HTA hub services. According to the definition provided above, HTAsiaLink is an HTA hub, and many HTA stakeholders in Asia and beyond perceive it to be operating in that capacity. For the purposes of this paper, references to Asia’s HTA hub refer to HTAsiaLink.

## Methods

A sequential, exploratory, mixed-method design was employed to assess the demand for HTA hub services in Asia (5). Technical and deliberative process dimensions of demand were explored, as were institutional aspects of how that demand could be met. Gaps identified during the literature review (stage 1) informed the design of the online survey instrument (stage 2). Information not found during the literature review and from survey results informed the development of the in-depth interview guides (stage 3).

### Stage 1: Literature review

The peer-reviewed literature and unpublished gray material were searched to define Asia’s current HTA landscape, along the dimensions shown in Table 1. Sources included organizational reports and publications related to HTA; journal article databases; and online web searches of HTA as a keyword in combination with Asia, Asian country and territory names, names of organizations of interest (i.e., those known to be involved in conducting, using, or supporting HTA in Asia), and topics relevant to HTA (e.g., capacity strengthening, institutionalization, and institutional development). Bibliographies and reference lists of material reviewed were searched to identify additional material.

**Table 1.** Topical dimensions of demand explored

Ensuring the supply of human resources needed to fully staff a health technology assessment (HTA) ecosystem
Institutional and systems development for country HTA ecosystems
Production and use of technical HTA output
Production of public goods
Financing HTA research and systems
Hub structure and technical support

### Stage 2: Survey of HTA stakeholders in Asia

Based on the literature review, a five-section, twenty-four item survey for online, anonymous self-administration was developed and sent to 125 Asia-focused HTA practitioners, including technical experts, HTA agency administrators, policymakers, healthcare providers, and advocates. Survey sections were: (i) respondent information; (ii) perceived need for HTA hub support in Asia (respondent’s organization); (iii) perceived need for HTA hub support in Asia (respondent’s country); (iv) demand for specific hub output; and (v) thoughts on a regional “home” for a regional HTA hub. The survey was administered in September 2022.

### Stage 3: In-depth interviews with HTA experts

Survey results were used to create in-depth interview guides. Starting with the list of 125 targeted survey respondents, 50 global and Asia regional HTA experts were purposively selected and invited to participate in 45–60 minute semi-structured interviews. Targeted key informants included HTA practitioners in Asia (including HTA “doers” and “users” representing government HTA agencies, HTA focal organizations, academic institutions, members of the HTAsiaLink Board of Directors, and technical assistance entities), global and regional funding partner organizations, and representatives from philanthropic foundations and global and regional organizations that support HTA development. Interviews were conducted between October and December 2022. To encourage frankness and openness by key informants, a statement was read at the start of each interview stating that participation was voluntary and that responses would be confidential.

Content analysis of interview output was performed in sequential steps. Interviews were transcribed, and content was abstracted into themes corresponding with components of the survey and the in-depth interview guides (Table 2). Sub-themes were identified for each content theme and organized by type of key informant. In the final stage, key messages were abstracted from each theme. Feedback on the analytic report was solicited from all key informants.

**Table 2.** Content analysis themes

Hub purpose and structure Priority programs and activities Engaging with hub members Hub financing Demand for a hub Technical support to the hub

Ethical approval (non-human subject research determination) was received from the MSH Scientific Committee.

## Results

### Literature review

Fifty-two peer-reviewed journal articles, twenty-one organizational reports, six slide decks, and three government documents in the formal and gray literature were identified and reviewed (Table 3). Nineteen Asian countries and territories were represented in the documents reviewed. Additional references pertained to HTA topics at the Asia regional and global levels.

**Table 3.** Summary of references reviewed during assessment stage 1

Reference topic	Types and number of references			
	Peer-reviewed articles	Slide decks	Organization reports	Government documents
Country HTA landscape	17	3	10	3
Organizational description or history	4	1	6	0
Regional landscape	3	1	1	0
Global HTA support	2	0	0	0
HTA challenges and needs	12		1	
HTA systems development and capacity building	5	1	0	0
Technical topic	9	0	3	0
Total reference documents reviewed	52	6	21	3

The literature provided a good basis for mapping Asia’s HTA institutional landscape. World Health Organization (WHO) country HTA profiles provided a general perspective on the state of HTA institutionalization (6;7). Several references described country-specific HTA landscapes (8–15); a larger group of references provided insight into the maturity and development needs from a cross-country perspective (1;2;16–25). A report on the 2022 proceedings of the HTA International (HTAi) Asia Policy Forum examined the region’s HTA capacity-strengthening needs (26). It explored the need for increased numbers of HTA workers, and for “upskilling” the current HTA workforce to keep pace with evolving HTA ecosystems and the increased demand for HTA evidence. Mundy and Maddern’s (27) article on the same forum drew attention to a need to mobilize sustainable financing for expanding HTA systems in the region.

The literature describes heterogeneous health and HTA systems in Asia, as well as variable application of HTA. Three tiers of HTA country systems are identifiable (28). The top tier includes countries with more mature health systems, where HTA application is widely practiced and HTA output is well embedded as a decision-making tool. The middle tier includes countries with an emergent HTA institutional base and where there is a strong commitment to using HTA output, especially to further universal health coverage (UHC) goals. In these countries, HTA output is still insufficient to meet growing demand. A third group of countries includes those where HTA application and using results in decision-making are mostly *ad hoc.*

The literature describes existing support for the development of technical HTA skills, such as economic evaluation methods and adaptive HTA (29), as well as efforts to foster HTA institutional development or to support deliberative processes in the HTA value chain (20;22;29). Several references provided detailed descriptions of country-level HTA development needs (13;14;30;31). While references on HTA hub financing are limited, existing literature on mission-driven non-profit organizations revolves around (a) risks and opportunities of diversified versus concentrated funding sources and (b) organizations’ ability to balance their mission focus with potentially misaligned funders’ priorities (32–36).

### Survey

**Figure 1.**
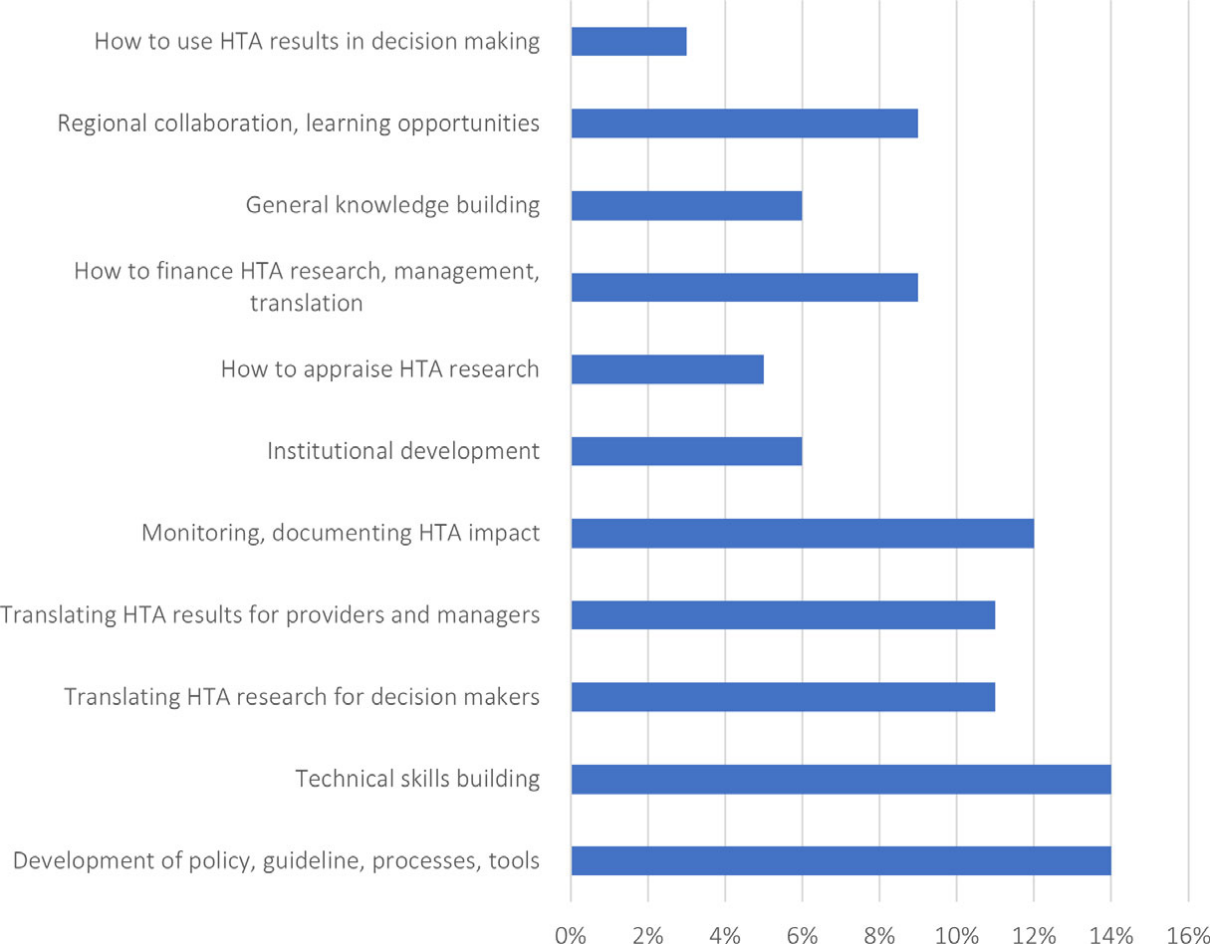
Survey results of the 140 instances where participants marked their top 3 areas of support based on their perceived organization or country needs.

Out of 125 recipients across twenty countries and territories, 25 (20 percent) recipients from ten countries and territories in Asia responded to the survey. Nineteen respondents (76 percent) rated their organization’s need for HTA strengthening as high or medium, and seventeen respondents (68 percent) rated their country’s need as high or medium. Categories of need expressed were distributed broadly across the range of response options provided (Figure 1). A regional institution was named nineteen times by survey respondents as an organization considered to be appropriate to provide such support; in fourteen of these instances, the respondents named HTAsiaLink, South Korea’s National Evidence-based Healthcare Collaborating Agency (NECA, HTAsiaLink’s past secretariat), or Thailand’s Health Intervention and Technology Assessment Program (HITAP, HTAsiaLink’s present secretariat).

Respondents were also asked to provide perspectives on desired HTA hub characteristics. The majority expressed that the regional hub should possess well-rounded knowledge and experience across the full spectrum of an HTA ecosystem. Named characteristics include strong HTA research and technical assistance experience as well as HTA institutionalization, policy development, and HTA decision-making experience. Respondents also expressed that having strong connections to international HTA networks and organizations is important and that the hub organization should be able to provide support in a manner that respects members’ current capacities and priorities.

### In-depth interviews

Out of the fifty targeted informants, twenty-eight (56 percent) accepted invitations to participate in in-depth interviews. Nineteen participated in individual interviews. In three instances, key informants chose to participate in small groups from their institutions (comprising nine key informants in total). Table 4 shows the countries, territories, and organizational types represented by key informants interviewed.

**Table 4.** Countries and organizational types represented in in-depth interviews

Country	Donor agencies	HTA practitioners in Asia	Implementing/technical assistance agencies	Other hubs	Total
Indonesia		1	1		2
Malaysia		3			3
Philippines		1			1
Singapore		1			1
South Korea		1			1
Taiwan		1			1
Thailand		5			5
Vietnam		1			1
Other	5		1	3	9
Multiple			4		4
Total	5	14	6	3	28

Abbreviation: HTA, Health Technology Assessment.

In-depth interview results provided additional evidence of strong demand for increased intra-regional HTA development support. Key informants perceive that HTAsiaLink and its secretariat already serve as Asia’s HTA hub. (South Korea’s NECA served in the secretariat role until 2022 after which Thailand’s HITAP assumed that role. HTAsiaLink’s by-laws allow for periodic change in the organization serving as its secretariat.) HTAsiaLink provides a widely recognized networking platform for regional HTA practitioners and organizations. They also connect the Asia HTA community to global HTA resources and platforms. Among the original study themes, three are discussed in detail: (i) priorities for strengthening HTA ecosystems in Asia, (ii) hub purpose, and (iii) hub financing.

## Discussion

Results show that demand in Asia for HTA hub support is high, and HTAsiaLink is widely supported to be that hub since it already seeks to respond to this demand. Three major themes are discussed below.

### Theme one: priorities for strengthening HTA ecosystems in Asia

This theme generated the most discussion by key informants. Four sub-themes emerged.

#### Sub-theme 1: Human resources capacity strengthening for HTA

Several academic institutions, government HTA organizations, and international technical assistance organizations active in Asia offer HTA-related short courses, on-the-job, and degree-oriented capacity strengthening. While a region-wide gap has not yet been quantified, it is generally perceived that the current supply of training opportunities likely falls short of the region’s rapidly increasing and diversifying needs for HTA professionals. Additionally, a broader focus on HTA training beyond HTA technical methodology is needed. Informants expressed a strong desire to see a coordinated, regional approach to HTA human resources capacity strengthening that augments existing, country-specific strategies. They recommended that country-level self-reliance in HTA training be included as a strategic goal for a regional plan. More broadly, key informants recommended that a regional partnership be created to produce an HTA human resources capacity-strengthening strategic plan and that partnership should include a regionally respected academic institution.

#### Sub-theme 2: Strengthened public goods

A range of HTA-related public goods exists in the region. These are products purposefully developed for the public to equitably access; they include HITAP’s Guide to Economic Analysis and Research Online Resource (GEAR) and a variety of reference case guides that help countries create national HTA agendas and navigate HTA institutionalization processes. There is strong demand in Asia for additional public goods to strengthen data and resource exchange systems and promote regional sharing of HTA materials, resources, and knowledge. For instance, the range of reference case materials could be broadened so that they cover all elements in an HTA ecosystem, including technical HTA production, appraisal, use of HTA output in decision-making, and impact evaluation of decisions made using HTA results. Key informants strongly endorse HTAsiaLink, with support from its secretariat, to lead the development and implementation of new public goods.

#### Sub-theme 3: Harmonization of HTA systems

Key informants to this study refer to harmonization as efforts to unify how HTAs are conducted and how results are used within and across countries. It refers to integration across a country HTA system’s elements (vertical harmonization), from setting HTA agendas and conducting HTAs to using HTA information in decision-making, implementation of those decisions, and evaluation of their impacts. Given the region’s diversity, the HTA hub could make a valuable contribution by defining a common understanding of HTA and how its outputs can be used. At the country level, a common understanding of HTA can promote efficient use of limited HTA resources by prioritizing assessments with the greatest potential to impact population health. Harmonization across countries (horizontal harmonization) can also promote efficiency by reducing research duplication through timely regional sharing of country-level HTA results. Based on reported experience of HTA hubs in other regions, key informants acknowledged that achieving harmonization is an ambitious challenge, but it is a potentially high-value activity for the hub.

#### Sub-theme 4: improved political will

Key informants expressed that political will for HTA in Asia needs to be strengthened to support HTA institutionalization and make better use of HTA output. To accomplish this, countries should seek support at senior health-sector leadership levels and among a broader range of allied senior-level policymakers, such as health insurance reimbursement agencies, government planning agencies, and finance ministries. While key informants acknowledged existing global initiatives that support strengthening political will, they expressed that the regional HTA hub would be the best vehicle for achieving regional ownership of these initiatives. To that end, regional and country-level dialogue should expand beyond the already-committed HTA community. Approaches could include incentivizing key policymakers to attend HTA conferences and dialogue forums as observers and presenters; creating space for key policymakers to establish their own forums to interact about how best to tap HTA’s value to health systems strengthening; increasing opportunities for patient, consumer, health advocacy, and other civil society groups to engage at all points along the HTA ecosystem continuum; and assisting public sector leaders to better engage private sector actors who influence political will for HTA. The goal would be to expand awareness about the potential value of HTA and to spur increased investment in HTA and use of its evidence. Advocacy tools such as investment case studies should be developed to build an evidence base of HTA impacts.

### Theme two: HUB purpose

There was general agreement among key informants that a hub organization derives legitimacy from responsiveness to members’ demands. Responsiveness can be communicated through a clear, transparent statement of organizational purpose. Key informants recommended that, given health and HTA systems diversity in Asia, the hub’s purpose should be grounded in a balance among serving *ad hoc* demands, country-specific needs, and longer-term regional goals. To define clear purposes and goals, key informants suggested that the hub engage in collaborative organizational strategic planning that taps the knowledge and strong commitment of HTAsiaLink’s members. As the region’s health systems and HTA ecosystems evolve, key informants also advised that the hub should periodically reengage members to map changes in needs and expectations.

Hub purpose and strategic goals could be defined around the priorities described in the previous sections. These priorities are not mutually exclusive, and key informants advised that hub purposes and goals link them to each other. For instance, building HTA human resource capacity without strengthening the political will for HTA and thus demand for HTA output among decision-makers might be inefficient and counterproductive. However, as a multidimensional purpose would require a more complex organizational and financing hub structure, key informants suggested that the hub could define a multidimensional purpose that phases in elements over defined medium- and long-term periods. This would allow the organization to build the technical, managerial, and financing base necessary to support an increasingly complex purpose and program.

Key informants expressed a strong preference for defining purpose, goals, strategies, and activities that the hub organization itself can implement and sustain. They advised cautious consideration of activities that require external technical consultants to implement and sustain. Anticipated challenges with external technical consultant support include path dependencies, limited retained capacity, and issues surrounding self-sufficiency. Key informants recommended that during activity implementation, the hub leverage the credibility and engagement of other respected institutions in the region. As a final note on this theme, key informants stressed the importance of defining a balance between staying focused on strategic plan implementation and being responsive to emergent and *ad hoc* needs, such as health emergencies and urgent country-specific policy needs.

### Theme three: HUB financing base and sources

Key informants consider country- and hub-level financing to be determining factors for ensuring the hub’s regional success. At the country level, there is the need to establish a dependable and sustainable financing base to support technical research, deliberative processes, and institutionalization aspects of the HTA ecosystem. Country-level financing needs to be in place for the hub to have a context for providing support. At the regional HTA hub level, there is a need to obtain and maintain financing for hub operations and programs. Required financing levels for the hub will change over time as they depend on the programs and services the hub offers and the pace at which it rolls them out.

This investigation identified three models for financing an HTA hub. RedETSA, the HTA hub serving Latin America and the Caribbean, is financed primarily through a combination of Pan American Health Organization (PAHO) support, where its secretariat sits, and grants from organizations in the region and beyond. Activities and programs of EUnetHTA, the HTA hub that served the EU, were cofinanced primarily by the EU and participating country governments. The International Network of Agencies for HTA (INAHTA), serving a global HTA community, represents a third approach; it is financed mostly by membership fees (37). For the Asia HTA hub, RedETSA’s funding partner-centered model is likely not feasible at this time given that the unique funding partner arrangement does not exist in Asia. Key informants expressed that having diversified funding sources would likely improve HTAsiaLink’s sustainability prospects. They described cofinancing (the EUnetHTA model) and member dues and fees (the INAHTA model) as currently having limited potential as stand-alone approaches in the Asia region, largely owing to the region’s diversity in the level of economic development and limitations on public finances. Key informants recommended that a regional initiative be undertaken to explore potential financing sources from a preliminary list and that a strategic resource mobilization plan be prepared. Potential sources include bilateral and multilateral funding partner agencies, global and regional philanthropic foundations, corporate social responsibility, public funding from member states, and hub-based member dues and fees. As each source presents differing prospects and challenges for sustainably financing and expanding hub activity, each one needs to be assessed for viability in the context of ensuring diversified financing sources.

Key informants further noted that alignment is needed between a hub’s structure and programs and how it is financed. A final concern arising out of in-depth interviews was the prospect of dynamic tension between the HTA hub’s need to be self-reliant in creating programs that serve regionally defined member needs and the goals and objectives of a potentially diverse range of funders. It is important for the hub to have a solid understanding of funders’ priorities and requirements and that it sets its own, clear boundaries.

### Strengths and weaknesses

This study possesses a number of strengths that enhance its robustness and applicability. A chief advantage lies in the sequential, exploratory mixed methods design involving a literature review, a survey, and in-depth interviews. Each stage built on and supplemented insights obtained from the previous stage. The initial literature review and survey stages provided a solid foundation of knowledge, which facilitated more informed, focused, and productive in-depth interviews. This sequential process provided an in-depth understanding of the HTA landscape in Asia. It also helped to identify topics for in-depth interviews and knowledgeable interview candidates. The interview guide was semi-structured to permit exploration of emergent themes not initially apparent from the first two stages. A further strength was the independent and confidential nature of the research process, intended to encourage candidness from key informants, even when discussing controversial issues within Asia’s closely connected HTA community. These strengths suggest a high degree of reliability and validity in the study’s findings.

The authors acknowledge several limitations to the present study. Despite the systematic literature review, publication bias may have impacted the range of accessed literature, potentially skewing the dataset toward more positive or significant results (38). Limitations inherent to online surveys, such as low response rates, response bias, and the potential lack of demographic representation (39), may have affected the reliability and generalizability of survey findings. While in-depth interviews were intended to correct for possible bias arising from the first two inquiry stages, the voluntary nature of in-depth interviews may have introduced its own selection bias. Some degree of nonrepresentativeness, inherent to the voluntary participation characteristic of qualitative research, in the final sample of key informants may have resulted in certain viewpoints having been overlooked. Additionally, private for-profit sector actors were not included in the pool of key informants; they may also hold valuable insights about how an HTA hub can add value to processes that promote HTA practice advancement and institutionalization.

Considering the results from this study and evolving regional HTA ecosystems, potential areas for future research include more in-depth exploration of hub organizational development, sustainability, and financing; assessment of emerging challenges and technical assistance needs; and evaluation of modes of support for HTA ecosystem advancement development.

## Conclusions

The findings of this study highlight a substantial demand for HTA technical, deliberative process, and institutional capacity strengthening in Asia. There is broad support for HTAsiaLink, in its capacity as the region’s HTA hub, to expand its role to meet this wide-ranging demand. Respondents identified a clear set of priorities for strengthening HTA ecosystems: developing an integrated regional plan for human resource capacity strengthening; augmenting and amplifying existing and new public goods; harmonizing HTA systems in the region; and improving the political will for HTA. They also stressed the importance that the hub’s organizational purpose respond to members’ demands, needs, and goals. Lastly, respondents expressed a concern for the sustainability of the hub, suggesting different models and approaches for a financing base and establishing more stable financing sources.

These findings underscore the importance of not only conducting HTA but also cultivating an environment that creates and fosters demand for HTA output. By leveraging existing commitment to HTA in the region, learning from accumulating experience and best practices, and responding to identified needs, the hub will continue to play a critical role in building HTA capacity and strengthening HTA ecosystems and, ultimately, in enhancing health outcomes across Asia. Continued and effective international collaboration is critical to the success of HTA hubs, and such is the case for HTAsiaLink as well.
